# Understanding host response to infectious salmon anaemia virus in an Atlantic salmon cell line using single-cell RNA sequencing

**DOI:** 10.1186/s12864-023-09254-z

**Published:** 2023-03-29

**Authors:** Ophélie Gervais, Carolina Peñaloza, Remi Gratacap, Athina Papadopoulou, Mariana Beltrán, Neil C. Henderson, Ross D. Houston, Musa A. Hassan, Diego Robledo

**Affiliations:** 1 The Roslin Institute and Royal (Dick) School of Veterinary Studies, University of Edinburgh, Midlothian, EH25 9RG UK; 2 Centre for Inflammation Research, the Queen´s Medical Research Institute, University of Edinburgh, Edinburgh, EH16 4TJ UK; 3 MRC Human Genetics Unit, Institute of Genetics and Cancer, University of Edinburgh, Crewe Road South, Edinburgh, UK; 4 Benchmark Genetics, 1 Pioneer Building, Edinburgh Technopole, Penicuik, EH26 0GB UK

**Keywords:** ISAV, Salmo salar, Single cell RNA-Seq, Aquaculture, SHK-1, Disease

## Abstract

**Background:**

Infectious Salmon Anaemia Virus (ISAV) is an Orthomixovirus that represents a large problem for salmonid aquaculture worldwide. Current prevention and treatment methods are only partially effective. Genetic selection and genome engineering have the potential to develop ISAV resistant salmon stocks. Both strategies can benefit from an improved understanding of the genomic regulation of ISAV pathogenesis. Here, we used single-cell RNA sequencing of an Atlantic salmon cell line to provide the first high dimensional insight into the transcriptional landscape that underpins host-virus interaction during early ISAV infection.

**Results:**

Salmon head kidney (SHK-1) cells were single-cell RNA sequenced at 24, 48 and 96 h post-ISAV challenge. At 24 h post infection, cells showed expression signatures consistent with viral entry, with genes such as PI3K, FAK or JNK being upregulated relative to uninfected cells. At 48 and 96 h, infected cells showed a clear anti-viral response, characterised by the expression of IFNA2 or IRF2. Uninfected bystander cells at 48 and 96 h also showed clear transcriptional differences, potentially suggesting paracrine signalling from infected cells. These bystander cells expressed pathways such as mRNA sensing, RNA degradation, ubiquitination or proteasome; and up-regulation of mitochondrial ribosome genes also seemed to play a role in the host response to the infection. Correlation between viral and host genes revealed novel genes potentially key for this fish-virus interaction.

**Conclusions:**

This study has increased our understanding of the cellular response of Atlantic salmon during ISAV infection and revealed host-virus interactions at the cellular level. Our results highlight various potential key genes in this host-virus interaction, which can be manipulated in future functional studies to increase the resistance of Atlantic salmon to ISAV.

**Supplementary Information:**

The online version contains supplementary material available at 10.1186/s12864-023-09254-z.

## Introduction

Aquaculture is currently the fastest growing food industry worldwide [1], and its products are a fundamental component of healthy and sustainable human diets. Atlantic salmon is the most important aquaculture fish species by value [1], and is a high-tech industry underpinned by large research and development programmes. Despite the high degree of technological progress, infectious diseases remain a major issue for salmon production. A notorious example is infectious salmon anaemia (ISA), a potentially fatal disease caused by virulent strains of the ISA virus (ISAV). ISAV is a fish Orthomyxovirus, and therefore, from the same family as Influenza viruses [2]. It is a segmented negative stranded RNA virus, with a genome that comprises 8 RNA segments coding for at least 10 proteins [3]. The clinical presentation of an ISAV infection is a severe anaemia, commonly accompanied by haemorrhage and necrosis of various organs [4]. ISA disease can cause up to 90% mortality in sea pens [5], and severe outbreaks have been responsible for the decimation of entire national aquaculture industries [4, 6].

Strategies to control and mitigate ISA varies across countries, although they mainly focus on disease containment [7]. As a notifiable pathogen, upon detection of a clinical ISAV outbreak, whole stocks have to be culled and the farm quarantined for a period of time [8]. Vaccines for ISA are available. Nevertheless, they do not offer complete protection and have proven insufficient to fully control the disease [9]. Encouragingly, resistance to ISA has been shown to be moderately heritable, which has been exploited by breeding companies to increase genetic resistance in salmon stocks based on regular experimental challenge testing of broodstock families and genomic selection [10–15]. While survival during such disease challenges is an important target trait for improvement, a detailed understanding of host-pathogen interactions holds great potential for fast tracking breeding efforts and the development of effective therapies against ISA. Firstly, it can assist in the identification of functional genetic variants to improve genomic selection accuracy and its persistency across distant relatives. Secondly, it can assist with the design of new vaccination, treatment, or management strategies. And thirdly, it can lead to targets for future genome editing studies to potentially develop fully ISA-resistant salmon strains.

Previous studies in Atlantic salmon have reported a notable up-regulation of the innate immune system in response to ISAV [15–18], albeit there is a large degree of tissue specificity in the responses [19]. This is expected since pathogen infections lead to complex and dynamic interactions with the host and its immune system. In vitro models provide simplified systems to study interactions between pathogens and the host cell machinery, which can help break down the multidimensional responses observed in vivo. The establishment of Atlantic salmon cell lines from various tissue types – e.g., gill, heart, and head kidney – has resulted in several in vitro models becoming available for the study of the main bacterial and viral diseases affecting salmon production [20–23]. In ISAV research, multiple in vitro studies have been performed, describing a rapid interferon response [24, 25] or the interplay between host and virus over the control of oxidative stress and apoptosis [26, 27]. In vitro studies using cell cultures have also been key to understand the role of the different ISAV proteins and their interaction with cellular mechanisms [28–31], further highlighting the importance of suitable model systems for the study of cell biology in Atlantic salmon.

While in vitro cell culture systems have led to key insights into cellular immune mechanisms and host-pathogen interactions, our understanding of ISA at the cellular and molecular level is still incomplete. One significant limitation of previous studies is that they were based on the analysis and interpretation of bulk RNA sequencing data, thus measured population-level cellular responses with limited resolution. In contrast, single-cell sequencing [32] allows us to obtain a more comprehensive picture of cellular heterogeneity, helping to unveil the complex dynamics of viral transcription and host molecular responses. Single cell sequencing has yet to be widely applied in Atlantic salmon, but has been used to gain insight into cellular transcription in gills [33]. Here, to better understand the molecular mechanisms of ISAV infection and the Atlantic salmon response to the virus, we have performed an in vitro ISAV infection in the salmon head kidney 1 (SHK-1) cell line. SHK-1 is a macrophage-like cell line that was established from adherent leukocytes isolated from the Atlantic salmon head kidney [34], the main hematopoietic organ in fish [35, 36]. Given SHK-1 is extensively used for ISAV research and viral diagnosis [26, 37–39], the high-resolution functional characterization of this cell line will contribute to build a rigorous model upon which to evaluate candidate targets or identify variants that may confer increased host resistance. The results of this study provide the first high-dimensional single-cell analysis of an ISAV infected fish cell line, revealing the heterogeneity of the infection process, and enabling the identification of early markers of disease and potential functional targets to impair ISAV infection.

## Methods

### Cell line

Atlantic salmon head kidney 1 (SHK-1) cells, an immortalised macrophage-like cell line derived from Atlantic salmon (*Salmo salar*), was obtained from the European Collection of Authenticated Cell Cultures (ECACC-97,111,106). The cells we obtained had been passaged 63 times, and the cells we used for the experiments were passaged 6 additional times for a total of 69 times. All cells were grown as a monolayer in L15 complete media (L15*), L15 (Sigma-Aldrich, St. Louis, USA) supplemented with 5% heat-inactivated foetal bovine serum (FBS) (Gibco, Waltham, USA), 40 µM β-mercaptoethanol (Gibco), 100 units/mL penicillin and 100 µg/mL streptomycin (Gibco). Cells were cultured in an incubator at 22 ± 1 °C without CO_2_. SHK-1 was split 1:2 at 80% confluency with 1/3 conditioned media.

### Viral stock

ISAV stock was obtained from Marine Research (Aberdeen, isolate V4782, derived from UK 2008/2009 outbreak). ISAV was passaged once on 1 x T175 SHK-1 cells (20 mL L15* but with reduced serum (2%) and the supernatant collected after 50% cell death, centrifuged, sterilised using a 0.45 μm filter) and stored in 1 mL aliquots at -80^ C^ until use. The viral dose was estimated by flow cytometry. To do this, SHK-1 cells were infected with different doses of the virus for 48 h in L15* with reduced serum (2%) at 17ºC, followed by antibody staining (ADL anti-ISAv, AquaMab-P10 and anti-mouse-GFP, Invitrogen, CA, #A21202) and fluorescence quantification (BD Fortessa X20).

### ISAV challenge

**Fig. 1:**
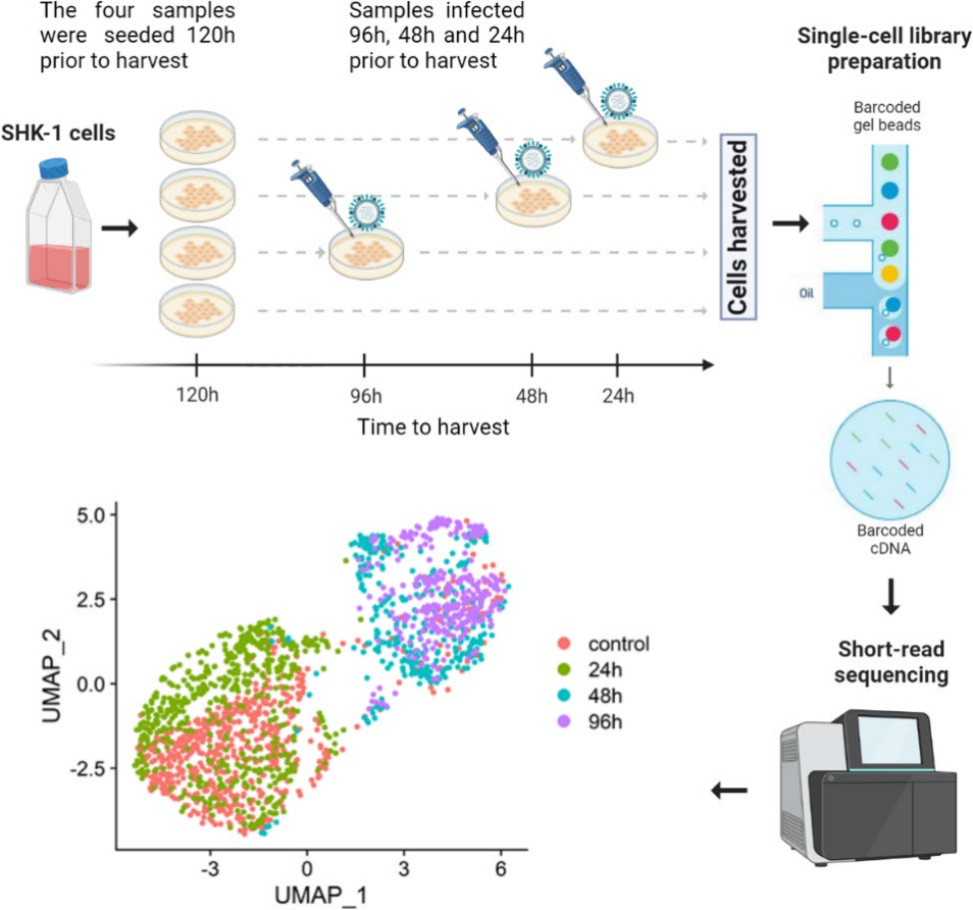
Experimental design and cell clustering. Diagrammatic figure showing the experimental design and workflow. Briefly, four SHK-1 cell plates were seeded at the same time, and three plates were infected 24 h, 48 and 96 h prior to harvesting. The four plates were collected 120 h post seeding, and immediately processed using the 10x workflow for single-cell RNA-seq library preparation. Libraries were sequenced using Illumina technology, and processed using Alevin and Seurat (see Methods) to cluster the cells of each sample according to their transcriptome

The SHK-1 cells were seeded at a density of 2.5 × 10^5^ SHK-1 in 6-well plates in L15 + 10% FCS and antibiotics (penicillin and streptomycin) 24 h prior to the challenge experiment. THE ISAV dose was determined using flow cytometry, and an ISAV infection dose of over 66% was used. The cells were left uninfected (control) or infected with 200 µL of ISAV in L15* with reduced serum (2%) and incubated at 17ºC for 24 h, 48 and 96 h. Cells (three infected time points and control) were collected by trypsin treatment, washed in phosphate buffered saline (PBS) and suspended to 10^6^ cells/mL in PBS + 0.05% BSA (Bio-Rad cell counter) (Fig. 1).

### Single-cell RNA sequencing library preparation and sequencing

Cells were counted and checked for viability on a Cell Counting Slide (Bio-Rad Laboratories Ltd) and then appropriately diluted according to 10x Guidelines. Each individual sample was loaded separately into the 10x Chromium machine and 10x single cell 3’ GEM kit v3 was used to generate the libraries following the manufacturer’s protocol. The quality of the resulting libraries was assessed in a Bioanalyzer (Agilent) and sequenced in two lanes of a NovaSeq SP flowcell with a cycle setup of 28/8/91 at Edinburgh Genomics.

### Single-cell RNA sequencing analysis

The raw single-cell RNA-seq samples control, [0 h (uninfected), 24 h, 48 and 96 h post-infection] were demultiplexed and mapped to a combined reference transcriptome of Atlantic salmon ICSASG_v2 (GCA_000233375.4) and ISAV (RefSeq, GCF_000854145.2) using Alevin/Salmon v1.4.0 [40]. The resulting raw count matrices were analysed using Seurat v3.1.5 [41] in R v3.6.3 [42]. Each library was loaded individually, discarding genes identified in fewer than 3 cells, and cells with fewer than 200 expressed genes. DoubletFinder V2.0.3 was used to assess the impact of doublets on cell clustering in each library, which was negligible. Thereafter all libraries were merged into a single Seurat object. For each cell, the percentage of mitochondrial and viral transcripts were estimated. After inspection of quality control parameters (Supplementary Fig. 1), cells with fewer than 1,000 or more than 10,000 genes or with percentage of mitochondrial transcripts above 25 were discarded. The infected/uninfected status of all cells was calculated as previously described [43]; based on the kernel density estimate of the distribution of percentage of viral counts on the log10 scale, which was used to find the first local minima. The percentage of viral transcripts in each cell was calculated using the “PercentageFeatureSet” function of Seurat using all viral genes. Cell cycle scores for each cell were estimated using the orthologues of Seurat´s (v3.1.5; Stuart at al. 2019) list of mammalian cell cycle markers. The gene counts of the filtered cells were normalised using ‘SCTransform’ with the percentage of mitochondrial RNA and cell cycle scores as regression variables. Dimensionality reduction was performed using the Uniform Manifold Approximation and Projection (UMAP) method using 30 principal components (determined using an Elbow plot; Supplementary Fig. 2), and clustered in groups using the ´FindNeighbors’ and ´FindClusters´ functions of Seurat (Stuart at al. 2019). Marker genes for each cell group were determined using the Wilcoxon Rank Sum test. Differentially expressed genes showing a log fold change > 0.25 and false discovery rate (FDR) corrected p-values < 0.05 were considered to be marker genes. Kyoto Encyclopedia of Genes and Genomes (KEGG) enrichment analyses were carried out using KOBAS v3.0.3 [44]. Briefly, salmon genes were annotated against the KEGG protein database [45] to determine KEGG Orthology (KO). KEGG enrichment for gene lists was tested by comparison to the whole set of expressed genes (obtained from the Seurat object) using Fisher’s Exact Test. KEGG pathways with ≥ 5 DE genes assigned and showing a Benjamini-Hochberg FDR corrected p-value < 0.05 were considered enriched. Finally, the correlation between viral and host genes was calculated. To compute the correlation of each viral gene with the whole salmon transcriptome, only the cells where that viral gene was expressed were selected. Person correlation between viral genes and the host genes was computed using the “cor” function in R, considering only correlation with r > |0.3|.

## Results and discussion

To assess the intracellular activity of ISAV and study the corresponding host response, single-cell RNA-Seq was performed on SHK cells infected with ISAV 24 h, 48 and 96 h post infection (Fig. 1). Uninfected SHK cells were used as controls. Raw reads were assigned to either Atlantic salmon genes or ISAV genes using a combined host-virus reference transcriptome. A total of 638, 512, 296 and 323 cells passed quality control filters for the control, 24 h, 48 and 96 h samples respectively. Clustering of the cells in these four libraries based on the host transcriptional response showed a clear separation between control and cells infected for 48 and 96 h. However, there was no transcriptional separation between control and cells infected for 24 h (Fig. 1).

### Viral transcription during infection

**Fig. 2:**
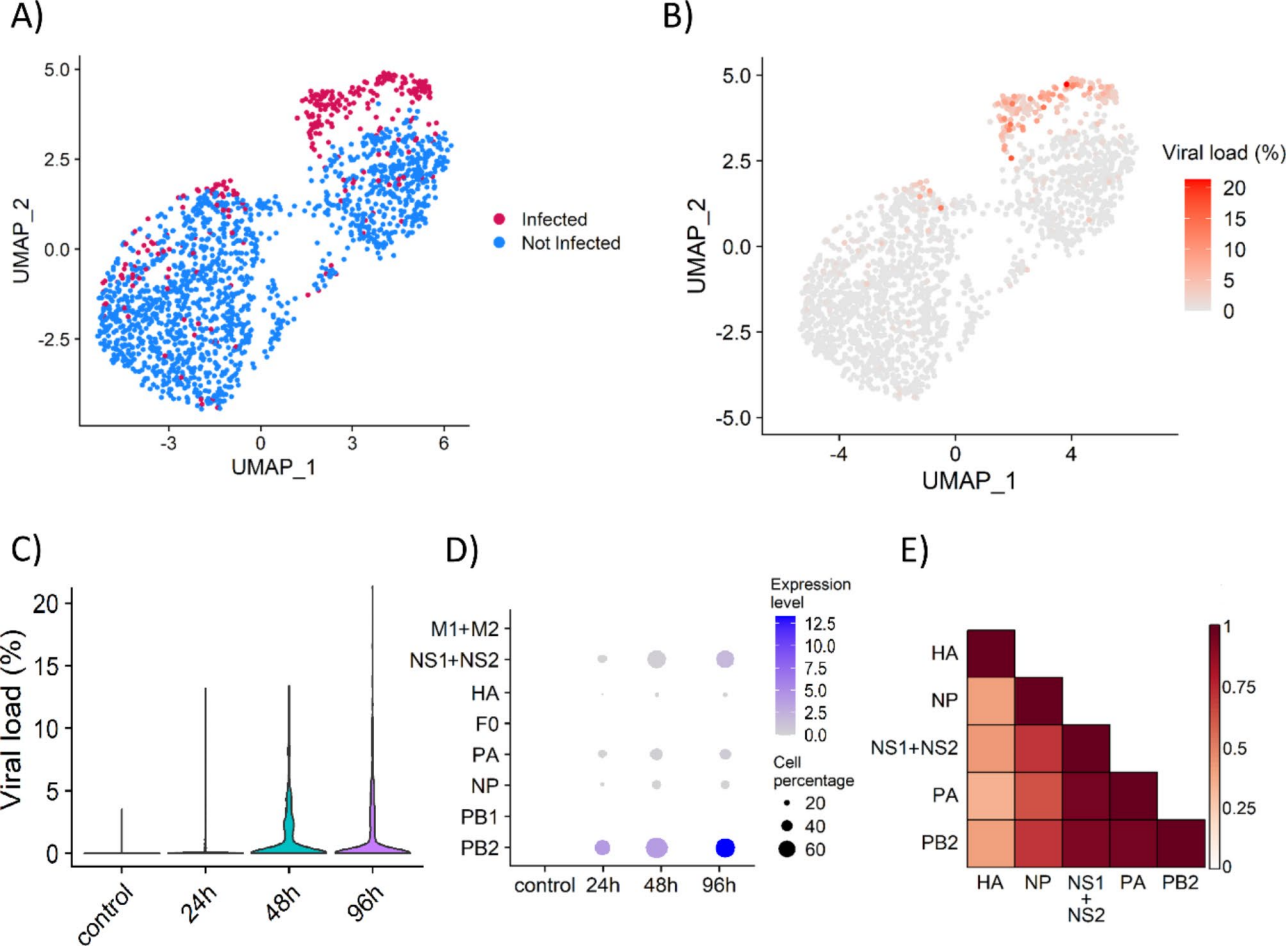
ISAV infection and transcription. (A) UMAP dimensionality reduction plot showing the infected and uninfected cells, where the threshold for infection was the local minima of the kernel density estimate of the distribution of percentage of viral counts on the log10 scale [43]. (B) UMAP dimensionality reduction plot showing the viral load of each cell measured as percentage of viral transcripts, calculated using all viral genes. (C) Viral load in each sample, measured as percentage of viral transcripts, calculated using all viral genes. (D) Dotplot showing the expression level, calculated as the percentage of the transcriptome of the cell that the viral genes represents, and the percentage of host cells with transcripts of each viral gene in each sample. (E) Heatmap showing the Pearson correlation between the expression of the viral genes

The percentage of infected cells (estimated following [43]) was 16% at 24 h post-infection, and 32–33% at 48 and 96 h (Fig. 2A). The infected cells formed two different groups, one with a small number of cells infected for 24 h, and the largest one with cells infected for 48 and 96 h where the percentage of viral transcripts was up to 22% of the transcriptome of the cells (Fig. 2B). Expectedly, the percentage of virus transcriptome, relative to the total cell transcriptome increased over the course of infection, mostly ranging between 2% and 15% (Fig. 2C). This is consistent with previous in vitro infections in other *Orthomyxoviridae*, although a larger heterogeneity has been described with viral transcript levels reaching up to 90% of the cell transcriptome [43, 46]. Viral transcript levels can vary substantially depending on the cell type [43], but also ISAV infection progression is typically slower than that of other *Orthomyxoviridae*, thus, later timepoints could show higher ISAV transcript levels.

The timing and level of expression of each viral gene also varies along the infection process (Fig. 2D). Of the 10 ISAV genes, 6 were found to be expressed in the cells. The viral polymerase basic protein 2 (PB2) gene is the most expressed throughout infection, followed by the polymerase acidic protein (PA) and the non-structural proteins 1 (NS1) and 2 (NS2), also known as nuclear exporting proteins or NEP (Fig. 2D). NS2 is a splicing product of NS1 [47] and is expressed at lower levels according to previous reports in ISAV [48] and Influenza A [49]. The expression of PB2, PA and NS2 showed a high positive correlation (0.92–0.96; Fig. 2E), suggesting their co-expression early during the infection, while NS1 showed a different expression pattern. Expression of the hemagglutinin (HA) is found in a small fraction of cells, and it shows the lowest correlation with the other (expressed) viral genes (Fig. 2E). Expression of the bicistronic mRNA encoding the matrix protein (M1) and a nuclear export protein (M2, also termed S8ORF2), of the polymerase basic 1 (PB1) and the viral membrane fusion protein (F0) was not detected in our dataset (Fig. 2D), which suggests they are either expressed late during the infection or expressed at low levels. In Influenza A, all the viral mRNAs show similar transcription patterns [50], however as mentioned above, ISAV infection typically progresses at a slower rate so it is possible that the viral mRNA dynamics are slightly different.

### Host response to ISAV

**Fig. 3:**
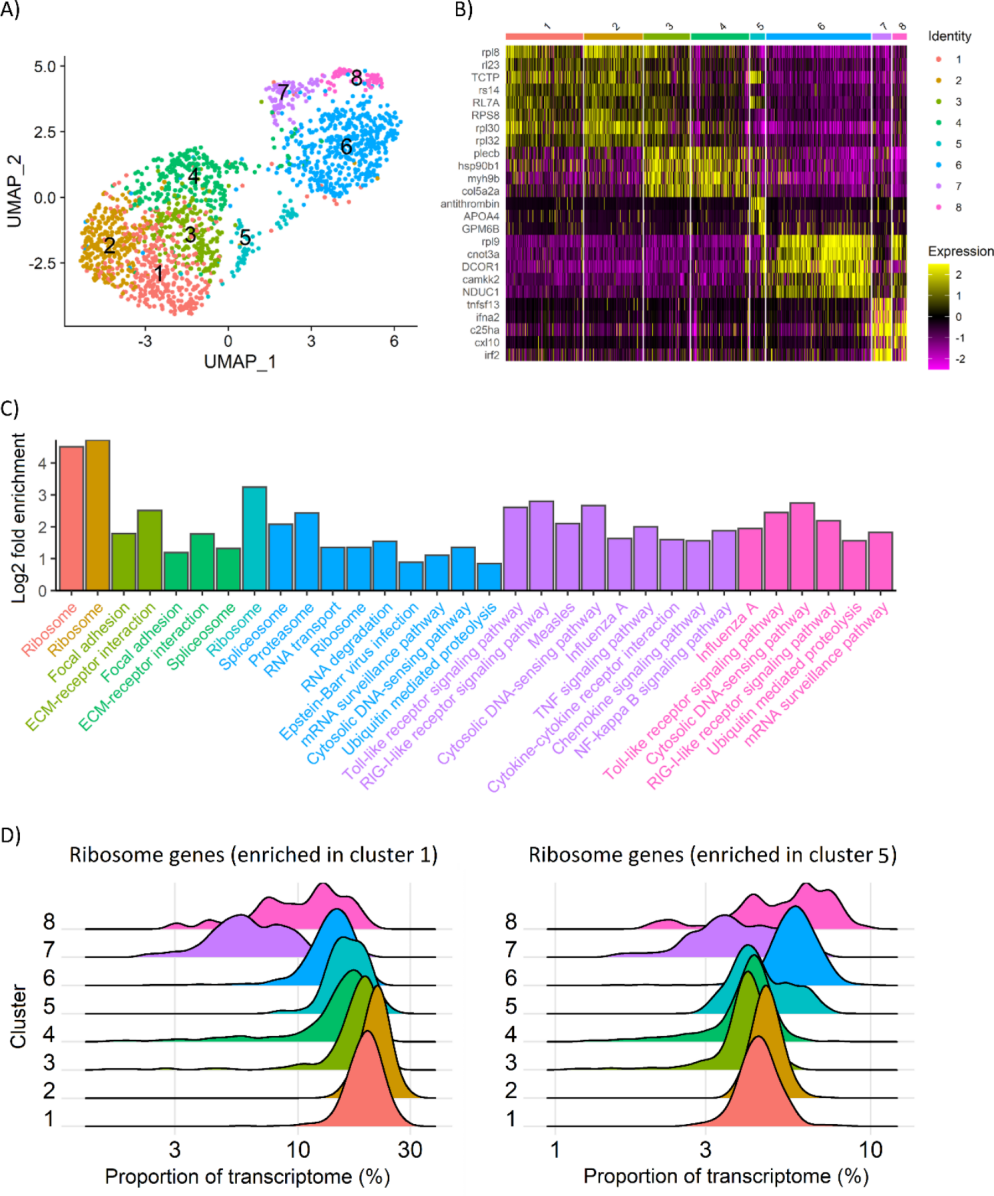
Response to ISAV in the Atlantic salmon SHK-1 cell line. (A) UMAP dimensionality reduction plot showing the clustering of the cells according to the host cell transcriptome; (B) Heatmap showing the expression pattern of the top marker genes for each cell cluster; (C) Barplot with the fold enrichment of selected KEGG pathways in each cell cluster, ordered within each cluster according to their significance; (D) Ridgeplot showing the proportion of the transcriptome that represent the two sets of “Ribosome” genes (KEGG pathway) enriched in (i) cluster 1 and (ii) cluster 5

The cells clustered into 8 different groups according to host gene expression (Fig. 3A). Despite working with a relatively homogeneous cell line, major transcriptional differences between the cells were linked to infection status and host response. Clusters 1 to 4 are formed by cell from the control and 24 h samples; 1, 2 and 3 are predominantly composed by cells from the control sample, but also have large numbers of 24 h cells (27.5 to 44.8% of the total cells in the cluster). On the contrary, cluster 4 is composed mostly of 24 h cells (82.5%), and a high proportion of those cells are infected with ISAV. On the other hand, cluster 6 is formed by 48 and 96 h uninfected cells, while cluster 7 and 8 are 48 and 96 h infected cells, respectively. The clear separation of control-24 and 48 h-96 h uninfected cells suggests paracrine signalling by infected cells may have caused altered gene expression in uninfected cells at the two latest time points. Paracrine signalling has been described before in response to Influenza A [51] and other viruses [52, 53], eliciting an antiviral interferon-mediated response in uninfected cells. We further tested this hypothesis by comparing control cells to uninfected cells at the infected timepoints (Supplementary File 1). The number of differentially expressed genes is quite high (835, 2607 and 3402 for control vs. 24 h, 48 and 96 h, respectively). However, our experimental design presents two potential caveats. First, the viral stock may contain some cytosolic content of death cells, and this could also have an effect on the cells transcriptome, indistinguishable of paracrine signalling; however, this potential cytosolic content does not seem to have an effect on the transcriptome of the cells at 24 h, and therefore we consider its effect to be negligible. Secondly, while we aimed for a relatively high infection rate, the results suggest that only a small fraction of cells were infected. It is therefore plausible that there are some reinfection events in the later timepoints. However, the apparently slow replication of the virus in our study (Fig. 2) and the fact that early infection does not seem to have a large impact on the transcriptome of the cells suggest that the observed changes in the transcriptome of non-infected cells at 48 and 96 h post infection should be in a great extent due to other causes. In any case, it is important to keep in mind these caveats in the interpretation of the later timepoints. Finally, cluster 5 is the most difficult to interpret since it is formed by a low number of cells of each sample apparently not connected to the infection process (Fig. 3A); this cluster could represent a different cell type since this cell line was originally reported to be heterogeneous [54], however we do not believe that is the case since our cells have undergone a high number of passages and they are homogenous under the microscope and are clustered together by flow cytometry.

Each of these clusters is characterised by specific marker genes (Fig. 3B, Supplementary File 2). Clusters 1 and 2 are characterised by the high expression of certain ribosomal genes, which also show a high expression in half of the cells of group 3. This could be either (i) an “artefact” of the cellular response in infected / bystander cells (high expression of additional genes leads to a lower relative expression of ribosomal genes), or (ii) due to manipulation of the ribosomal machinery by ISAV, which can be connected to the hijacking of host translational machinery by the virus, a common strategy used by viruses to repress the cellular mRNA translation and allow the preferential translation of viral mRNAs [55]. On the other hand, a group of immune genes, including interferon alpha 2 (*ifna2*) or interferon regulatory factor 2 (*irf2*), are highly expressed in infected cells at 48 h (cluster 7), but moderately at 96 h (cluster 8), suggesting either viral repression or a shift in the immune response later during the infection. Further, this group of genes is not highly expressed in bystander cells at 48 and 96 h (cluster 6), suggesting that paracrine signalling by infected cells does not promote the activation of an interferon response in bystander cells.

To facilitate an unbiased interpretation of the marker genes of each cluster we performed a KEGG pathway enrichment analysis (Fig. 3C, Supplementary File 3). This confirmed the enrichment in ribosomal genes in the control / uninfected clusters (1 and 2). Clusters 3 and 4 (mostly 24 h cells, particularly in cluster 4) are enriched for “focal adhesion” and “ECM-receptor interactions”, which may reflect a consequence of the interaction of ISAV with the extracellular matrix and membrane receptors. The genes in these pathways include phosphoinositide 3-kinase (*pi3k*), focal adhesion kinase (*fak*) and c-Jun terminal kinase (*jnk*), which have been shown to be important for the entry of Influenza A into the cytoplasm [56–58]. This is consistent with the similarity in the entry mechanisms of Influenza A and ISAV [39, 59] and suggests that molecular interactions during viral entry are also conserved to some extent. These early genes are good candidates for functional studies aimed at disrupting ISAV entry into salmon cells and eventually to develop strategies to tackle ISAV in Atlantic salmon aquaculture, including the development of genome edited ISAV-resistant stocks.

Uninfected bystander cells at 48 and 96 h (cluster 6) showed enrichment for terms such as “proteasome”, “RNA degradation”, “mRNA surveillance pathway” or “cytosolic DNA-sensing pathway”, but they do not express typical immune genes (i.e. interferon or cytokines). This cluster shows expression of the seven *lsm* genes that compose the LSm1-7 ring involved in the degradation of messenger RNA in the cytoplasm [60]. The host RNA decay machinery can rapidly degrade viral mRNAs, and as such viruses have developed strategies to avoid or even use to their advantage parts of this pathway, including the LSm1-7 ring [61]. Many proteasome-specific genes are also expressed in this cluster, including two of the subunits of the immunoproteasome, involved in the degradation of intracellular proteins, including those of viral origin for presentation on major histocompatibility complex [62]. Again, many virus interact with the immunoproteasome as part of their infection strategy [63]. Connected to the proteasome, this cluster also expresses all the genes of the ubiquitination machinery: E1 ubiquitin-activating enzymes, E2 ubiquitin-conjugating enzymes and E3 ligases of all the types (HERC3, U-box and RING-finger and their adaptor proteins). Up-regulation of genes related to ubiquitination has been previously described in resistant fish in response to ISAV [64]. Moreover, proteins encoded by ISAV segment 8 (M1 and M2), which are known to interfere with interferon signalling, can be conjugated to ubiquitin and the ubiquitin-like interferon simulated gene 15 (ISG15) in Atlantic salmon cells [65]. Several viruses can hijack the host ubiquitination machinery, and in particular Influenza uses host ubiquitination as part of its cell entry and replication strategy [66–68]. Ubiquitination is also a fundamental part of the host immune response, controlling multiple cellular processes, and its role in the activation of the interferon response via TRIM25 - RIG-I interaction is well-documented [69]. Ubiquitination has been barely studied in fish; considering the large expansion of TRIM E3 ubiquitin ligases in fish [70], our recent results and the evidence in other species, ubiquitination deserves more attention to try to understand ISAV infection in Atlantic salmon.

Bystander cells at 48 and 96 h (cluster 6) also showed enrichment in ribosomal genes, as control and cells infected for 24 h (cluster 1 and 2), which was unexpected based on the expression pattern of ribosomal genes in the heatmap (Fig. 3B). However, we found that the underlying specific ribosomal genes are different, with only two genes in common between the two groups of cells (out of 55 in each list). The ribosomal genes enriched in Clusters 1 and 2 showed lower expression in the infected cells at 48 and 96 h (clusters 7 and 8), while the ribosomal genes enriched in Cluster 6 showed high expression also in infected cells at 96 h (cluster 8), but not so clearly in 48 h infected cells (cluster 7), although a wider expression level distribution was observed in these cells (Fig. 3D). A closer look at the gene lists revealed that most of the ribosomal genes more expressed in clusters 6 and 8 (48 h + 96 h bystander cells and 96 h infected cells) code for proteins of the mitochondrial ribosome (Supplementary file 2). Mitochondria activate anti-viral immune responses through mitochondrial antiviral signalling (MAVS) [71] and can also initiate apoptosis [72]. Influenza A NS1 protein has been observed in the mitochondria [73], and is able to alter mitochondria morphodynamics [74]. Our results suggests that paracrine regulation upon ISAV infection involves the mitochondria as a defence mechanism, and the fact that these mitochondria ribosomal genes are also overexpressed at 96 h in infected cells (Fig. 3D, cluster 8) suggests that they may play an active role in the immune response against ISAV.

Finally, clusters 7 and 8 (infected 48 and 96 h cells, respectively) show up-regulation of key immune response genes, such as interferon alpha 2 and 3 (*ifna2*, *ifna3*), interferon regulatory factor 2 (*irf2*) or C-X-C motif chemokine 10 (*cxl10*). These genes are slightly more up-regulated in the 48 h than in the 96 h cells (e.g. logFC 3.7 vs. 1.7 for *ifna3*, 3.8 vs. 1.6 for ifna2; Supplementary File 2), with pathways such as toll-like receptor signalling, RIG-I receptor signalling, TNF signalling or cytokine-cytokine interaction. However, these genes were not expressed in bystander cells at 48 and 96 h (cluster 6). Our results align with previous studies that have shown that ISAV triggers an early immune response characterised by the activation of interferon genes, both in vitro and in vivo [19, 75, 76]. IFNA2, IFNA3 and CXL10 are secreted proteins that act on other cells to promote an antiviral state and could explain the “activated” state of cluster 6 cells. However, surprisingly, they did not induce the up regulation of interferon genes in these cells, which is quite different to what has been reported during Influenza infections, where the paracrine signalling is important at 12 h post infection and increases the expression of interferon stimulated genes in bystander cells [51]. There are different potential explanations for this finding, for instance the virus may be able to inhibit the interferon-related paracrine signalling quite early during the infection, but it is also plausible that paracrine signalling functions differently in fish than in mammals. Further investigations are necessary to understand fish paracrine signalling, including studies with other fish species and viruses.

### Interactions between host and viral genes

**Fig. 4:**
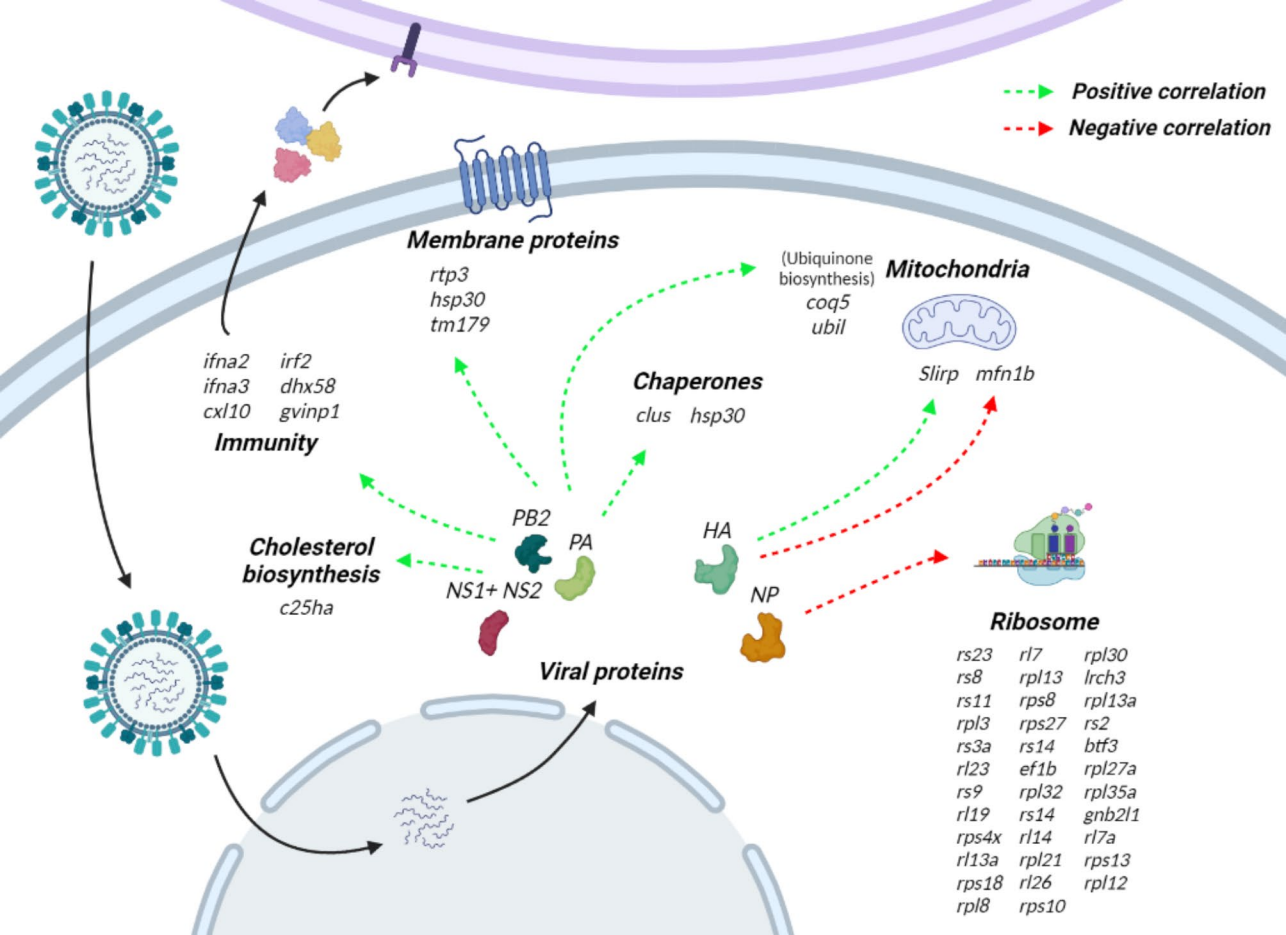
Correlation between viral gene expression and host gene expression. All Pearson correlation values are between |0.3| and |0.5| (Supplementary File 4)

To evaluate whether specific viral proteins could have a direct impact on the expression of host genes, a correlation analysis was performed between expressed viral and host genes in infected cells (Fig. 4, Supplementary file 4). No strong correlations were observed (highest r = 0.51) and only those correlations with r > |0.3| were considered. *PB2*, *PA* and *NS1 + NS2* expression patterns showed significant correlation with largely the same set of host genes. Considering these viral genes are the most expressed, these correlations do not necessarily represent regulation of host genes by viral proteins; they could also represent genes that respond proportionally to viral load. In fact, the host genes exhibiting the highest positive correlation include the immune genes mentioned in the previous section (*ifna2, ifna3, irf2*) and the regulator of antiviral signalling probable ATP-dependant RNA helicase DHX58 (*dhx58*). The highest correlation was observed with cholesterol 25-hydroxylaseI A (*c25ha*), an interferon-stimulated gene involved in the regulation of cholesterol biosynthesis, which has broad antiviral activity [77]. There were also host genes showing negative correlations with these viral genes, especially *NS1 + NS2*, for example with Phosphoinositide-3-Kinase Regulatory Subunit 1 (*pik3r1*), which may be exploited to tackle ISAV infections; Influenza A NS1 protein modulates the PIK3 / Akt pathway via interaction with PIK3, and disrupting this interaction led to reduced infectivity [78]. Several transcription factors also showed a negative correlation with *NS1 + NS2*, such as twist-related protein 2 (*twst2*) or zinc-finger protein SNAI1 (*snai1*).

The expression of over thirty cytoplasmic ribosomal genes is negatively correlated with the expression of the viral nucleoprotein NP (r = -0.3 to -0.4), suggesting a negative regulation of the host translation machinery by the virus. While NP is a structural protein, it has been shown that it interacts with host factors to facilitate viral replication [79, 80], although no interactions between NP and ribosomal genes have been previously described. The ribosomal machinery is an important part of viral replication since viral genomes do not usually harbour mRNA translation genes [81], and therefore they need to recruit host proteins including ribosomal proteins (RPs) to complete their replication cycle. Our results show that the viral protein NP may interact or at least drive the expression of specific mitochondrial RPs. While it is well known that viruses interact with ribosomal proteins as part of their infection strategy [82, 83], recent studies have also highlighted that some ribosomal proteins can have an antiviral function by either interacting with viral proteins to inhibit transcription/translation or by activating antiviral defence signalling pathways [82]. Non-infected cells in 48 and 96 h infected samples showed a clear up-regulation of mitochondrial RPs, which could suggest that they form an important part of a potential antiviral pathway in bystander cells, which may be repressed by the viral protein NP in infected cells considering the negative correlation.

There were also several chaperons showing positive correlation with viral genes, such as host heat shock protein 30 (hsp30) and clusterin (*clu*), which has antiapoptotic activity and is directly targeted by the Influenza A virus nucleoprotein [84]. Chaperones are used by viruses to facilitate the folding and assembly of the viral proteins [85]. Another protein of interest is sequestosome-1 (*sqstm1*), a receptor required for selected autophagy that inhibits Seneca valley virus and avian influenza replication [86, 87]; previous studies have reported that mammalian cells can use autophagy to restrict the replication of avian Influenza [88], but it has also been reported that it can promote the replication of influenza A [89, 90]. Sqstm1 is also known as ubiquitin-binding protein p62, being able to bind ubiquitin and providing the link between ubiquitination (relevant in bystander and infected cells; Fig. 3C and Supplementary File 2) and autophagy [73].

While it is not possible to discriminate whether the observed correlations between viral and host proteins are caused by (i) proportional response of the host to viral load or (ii) regulation of the host transcription by the virus, the genes and processes highlighted help us better understand the infection process and represent good targets for further functional studies aiming to develop ISAV-resistant Atlantic salmon. We have also identified genes without previous known associations to viral processes, such as the transcription factors *twst2* or *snai1*. In vitro functional analyses are required to confirm the potential of these genes to sway the salmon-ISAV interaction in the fish favour (e.g. via disruption of their interactions with viral proteins), paving the way for their application in aquaculture to limit the impact of this disease and improving fish welfare and food security.

## Conclusion

Here we applied single-cell RNA sequencing to study ISAV infection in Atlantic salmon SHK-1 cells. This simplified system, in combination with this novel genomics technology, has allowed us to finely characterise the transcriptomic changes that occur during early ISAV infection both in the virus and the host, and suggest potential interactions. Early ISAV infection of Atlantic salmon SHK cells is characterised by high expression of the PB2, NS1 + NS2 and PA viral genes, which show highly correlated expression patterns. After 24 h of infection infected SHK cells showed similar transcriptomic profiles to control cells, however genes and pathways connected to viral entry were identified, which could be good targets for functional studies to impair viral infection. At 48 and 96 h post-infection there was a clear host transcriptomic response to the virus which also affected uninfected cells, therefore suggesting paracrine signalling from infected cells. This putative paracrine signalling seemed to produce an unspecific alert state in the uninfected cells, involving mRNA surveillance and RNA degradation, ubiquitination and the proteasome, or mitochondrial activation. These pathways were also up-regulated in 48 and 96 h infected cells, but they also showed a clear antiviral interferon response, including genes such as *ifna* or *irf2*. Correlation between the expression of viral and host genes suggested potential negative regulation of certain host transcription factors and ribosomal genes by viral proteins. This study increases our understanding of the molecular interactions between ISAV and Atlantic salmon cells, and provides new targets for functional studies aiming to increase the resistance of Atlantic salmon stocks, leading to increased food security and fish welfare.

## Electronic supplementary material

Below is the link to the electronic supplementary material.

Supplementary Material 1

Supplementary Material 2

Supplementary Material 3

Supplementary Material 4

Supplementary Material 5

Supplementary Material 6

Supplementary Material 7

## Data Availability

Single-cell RNA sequencing raw reads have been deposited in NCBI’s Sequence Read. Archive (SRA) under BioProject accession number PRJNA878763 (https://www.ncbi.nlm.nih.gov/bioproject/PRJNA878763).

## References

[1] FAO. 2020. The State of World Fisheries and Aquaculture 2020. Sustainability in action. Rome. 10.4060/ca9229en.

[2] Toennessen R, Lauscher A, Rimstad E (2009). Comparative aspects of infectious salmon anemia virus, an orthomyxovirus of fish, to influenza viruses. Indian J Microbiol.

[3] Rimstad E, Markussen T (2020). Infectious salmon anaemia virus—molecular biology and pathogenesis of the infection. J Appl Microbiol.

[4] Aamelfot M, Dale OB, Falk K (2014). Infectious salmon anaemia – pathogenesis and tropism. J Fish Dis.

[5] Dannevig BH, Mjaaland S, Rimstad E. Infectious Salmon Anemia Virus. In: Mahy BWJ, Van Regenmortel MHV, editors. Encyclopedia of Virology (Third Edition). Oxford: Academic Press; 2008. p. 89–95.

[6] Godoy MG, Aedo A, Kibenge MJT, Groman DB, Yason CV, Grothusen H (2008). First detection, isolation and molecular characterization of infectious salmon anaemia virus associated with clinical disease in farmed Atlantic salmon (Salmo salar) in Chile. BMC Vet Res.

[7] Aldrin M, Huseby RB, Bang Jensen B, Jansen MD (2021). Evaluating effects of different control strategies for infectious Salmon Anaemia (ISA) in marine salmonid farming by scenario simulation using a disease transmission model. Prev Vet Med.

[8] OIE-Listed diseases. OIE - World Organisation for Animal Health; 2020 [cited 2022 Aug 7]. Database: Animal Diseases [Internet]. Available from: https://www.oie.int/en/animal-health-in-the-world/oie-listed-diseases-2020/.

[9] Kibenge FS, Munir K, Kibenge MJ, Joseph T, Moneke E (2004). Infectious salmon anemia virus: causative agent, pathogenesis and immunity. Anim Health Res Rev.

[10] Gjøen HM, Bentsen HB (1997). Past, present, and future of genetic improvement in salmon aquaculture. ICES J Mar Sci.

[11] Ødegård J, Olesen I, Gjerde B, Klemetsdal G (2007). Evaluation of statistical models for genetic analysis of challenge-test data on ISA resistance in Atlantic salmon (Salmo salar): prediction of progeny survival. Aquaculture.

[12] Kjøglum S, Henryon M, Aasmundstad T, Korsgaard I (2008). Selective breeding can increase resistance of Atlantic salmon to furunculosis, infectious salmon anaemia and infectious pancreatic necrosis. Aquac Res.

[13] Gjerde B, Evensen Ø, Bentsen HB, Storset A (2009). Genetic (co)variation of vaccine injuries and innate resistance to furunculosis (Aeromonas salmonicida) and infectious salmon anaemia (ISA) in Atlantic salmon (Salmo salar). Aquaculture.

[14] Holborn MK, Ang KP, Elliott JAK, Powell F, Boulding EG (2020). Genome wide analysis of infectious salmon anemia resistance in commercial Saint John River Atlantic salmon. Aquaculture.

[15] Gervais O, Barria A, Papadopoulou A, Gratacap RL, Hillestad B, Tinch AE (2021). Exploring genetic resistance to infectious salmon anaemia virus in Atlantic salmon by genome-wide association and RNA sequencing. BMC Genomics.

[16] Jørgensen SM, Afanasyev S, Krasnov A (2008). Gene expression analyses in Atlantic salmon challenged with infectious salmon anemia virus reveal differences between individuals with early, intermediate and late mortality. BMC Genomics.

[17] Lauscher A, Krossøy B, Frost P, Grove S, König M, Bohlin J (2011). Immune responses in Atlantic salmon (Salmo salar) following protective vaccination against infectious salmon anemia (ISA) and subsequent ISA virus infection. Vaccine.

[18] Dettleff P, Moen T, Santi N, Martinez V (2017). Transcriptomic analysis of spleen infected with infectious salmon anemia virus reveals distinct pattern of viral replication on resistant and susceptible Atlantic salmon (Salmo salar). Fish Shellfish Immunol.

[19] Valenzuela-Miranda D, Boltaña S, Cabrejos ME, Yáñez JM, Gallardo-Escárate C (2015). High-throughput transcriptome analysis of ISAV-infected Atlantic salmon Salmo salar unravels divergent immune responses associated to head-kidney, liver and gills tissues. Fish Shellfish Immunol.

[20] Gjessing MC, Aamelfot M, Batts WN, Benestad SL, Dale OB, Thoen E (2018). Development and characterization of two cell lines from gills of Atlantic salmon. PLoS ONE.

[21] Heidrun IW, Ragnhild Aakre J (2001). A salmonid cell line (TO) for production of infectious salmon anaemia virus (ISAV). Dis Aquat Organ.

[22] Fryer JL, Lannan CN (1994). Three decades of fish cell culture: a current listing of cell lines derived from fishes. J Tissue Cult Methods.

[23] Zúñiga A, Aravena P, Pulgar R, Travisany D, Ortiz-Severín J, Chávez FP, et al. Transcriptomic changes of Piscirickettsia salmonis during Intracellular Growth in a Salmon Macrophage-Like Cell line. Front Cell Infect Microbiol. 2020;9. 10.3389/fcimb.2019.00426. 10.3389/fcimb.2019.00426 PMC6964531 31998656

[24] Andresen AMS, Boudinot P, Gjøen T (2020). Kinetics of transcriptional response against poly (I:C) and infectious salmon anemia virus (ISAV) in Atlantic salmon kidney (ASK) cell line. Dev Comp Immunol.

[25] Samsing F, Hoad J, Mohr P, Dearnley M, Wynne JW (2020). Comparative transcriptome analysis of pilchard orthomyxovirus (POMV) and infectious salmon anaemia virus (ISAV). Fish Shellfish Immunol.

[26] Olavarría VH, Recabarren P, Fredericksen F, Villalba M, Yáñez A (2015). ISAV infection promotes apoptosis of SHK-1 cells through a ROS/p38 MAPK/Bad signaling pathway. Mol Immunol.

[27] Olavarría VH, Valdivia S, Salas B, Villalba M, Sandoval R, Oliva H (2015). ISA virus regulates the generation of reactive oxygen species and p47phox expression in a p38 MAPK-dependent manner in Salmo salar. Mol Immunol.

[28] Li C, Greiner-Tollersrud L, Robertsen B (2016). Infectious salmon anemia virus segment 7 ORF1 and segment 8 ORF2 proteins inhibit IRF mediated activation of the Atlantic salmon IFNa1 promoter. Fish Shellfish Immunol.

[29] Zhang W, Cai C, Lin L, Tao YJ, Jin M (2017). Subcellular localization and interactions of infectious Salmon Anemia Virus (ISAV) M1 and NEP as well as host Hsc70. Virol J.

[30] Thukral V, Varshney B, Ramly RB, Ponia SS, Mishra SK, Olsen CM (2018). s8ORF2 protein of infectious salmon anaemia virus is a RNA-silencing suppressor and interacts with Salmon salar Mov10 (SsMov10) of the host RNAi machinery. Virus Genes.

[31] Toro-Ascuy D, Santibañez A, Peña V, Beltran-Pavez C, Cottet L, Molina C (2020). Development of an Isavirus minigenome system to study the function of the pocket RNA-binding domain of the viral nucleoprotein (NP) in salmon cells. J Fish Dis.

[32] Tang F, Barbacioru C, Wang Y, Nordman E, Lee C, Xu N (2009). mRNA-Seq whole-transcriptome analysis of a single cell. Nat Methods.

[33] West AC, Mizoro Y, Wood SH, Ince LM, Iversen M, Jørgensen EH (2021). Immunologic profiling of the Atlantic Salmon Gill by single nuclei transcriptomics. Front Immunol.

[34] Dannevig BH, Falk K, Namork E (1995). Isolation of the causal virus of infectious salmon anaemia (ISA) in a long-term cell line from Atlantic salmon head kidney. J Gen Virol.

[35] Neumann NF, Barreda D, Belosevic M (1998). Production of a macrophage growth factor(s) by a goldfish macrophage cell line and macrophages derived from goldfish kidney leukocytes. Dev Comp Immunol.

[36] Joerink M, Ribeiro CM, Stet RJ, Hermsen T, Savelkoul HF, Wiegertjes GF (2006). Head kidney-derived macrophages of common carp (Cyprinus carpio L.) show plasticity and functional polarization upon differential stimulation. J Immunol.

[37] Rolland JB, Bouchard D, Coll J, Winton JR (2005). Combined use of the ASK and SHK-1 cell lines to enhance the detection of infectious salmon anemia virus. J Vet Diagn Invest.

[38] Joseph T, Cepica A, Brown L, Ikede BO, Kibenge FSB (2004). Mechanism of cell death during infectious salmon anemia virus infection is cell type-specific. J Gen Virol.

[39] Eliassen TM, Frøystad MK, Dannevig BH, Jankowska M, Brech A, Falk K (2000). Initial events in infectious salmon anemia virus infection: evidence for the requirement of a low-pH step. J Virol.

[40] Srivastava A, Malik L, Smith T, Sudbery I, Patro R (2019). Alevin efficiently estimates accurate gene abundances from dscRNA-seq data. Genome Biol.

[41] Stuart T, Butler A, Hoffman P, Hafemeister C, Papalexi E, Mauck WM (2019). Comprehensive Integration of single-cell data. Cell.

[42] R Core Team (2018). R: a language and environment for statistical computing.

[43] Sun J, Vera JC, Drnevich J, Lin YT, Ke R, Brooke CB (2020). Single cell heterogeneity in influenza a virus gene expression shapes the innate antiviral response to infection. PLoS Pathog.

[44] Xie C, Mao X, Huang J, Ding Y, Wu J, Dong S (2011). KOBAS 2.0: a web server for annotation and identification of enriched pathways and diseases. Nucleic Acids Res.

[45] Kanehisa M, Goto S (2000). KEGG: kyoto encyclopedia of genes and genomes. Nucleic Acids Res.

[46] Russell AB, Trapnell C, Bloom JD (2018). Extreme heterogeneity of influenza virus infection in single cells. eLife.

[47] Ramly RB, Olsen CM, Braaen S, Rimstad E (2013). Infectious salmon anaemia virus nuclear export protein is encoded by a spliced gene product of genomic segment 7. Virus Res.

[48] Ramly RB, Olsen CM, Braaen S, Hansen EF, Rimstad E (2014). Transcriptional regulation of gene expression of infectious salmon anaemia virus segment 7. Virus Res.

[49] Robb NC, Jackson D, Vreede FT, Fodor E (2010). Splicing of influenza a virus NS1 mRNA is independent of the viral NS1 protein. J Gen Virol.

[50] Phan T, Fay EJ, Lee Z, Aron S, Hu W-S, Langlois RA (2021). Segment-specific kinetics of mRNA, cRNA, and vRNA accumulation during influenza infection. J Virol.

[51] Ramos I, Smith G, Ruf-Zamojski F, Martínez-Romero C, Fribourg M, Carbajal EA (2019). Innate immune response to influenza virus at single-cell resolution in human epithelial cells revealed paracrine induction of interferon Lambda 1. J Virol.

[52] Patil S, Fribourg M, Ge Y, Batish M, Tyagi S, Hayot F (2015). Single-cell analysis shows that paracrine signaling by first responder cells shapes the interferon-β response to viral infection. Sci Signal.

[53] Voigt EA, Swick A, Yin J (2016). Rapid induction and persistence of paracrine-induced cellular antiviral states arrest viral infection spread in A549 cells. Virology.

[54] Dannevig BH, Falk K, Namork E. Isolation of the causal virus of infectious salmon anaemia (ISA) in a long-term cell lines from Atlantic salmon head kidney.J Gen Virol. 1995:1353–1359. doi 10.1099/0022-1317-76-6-1353 7782764

[55] Levene RE, Gaglia MM (2018). Host shutoff in influenza a virus: many means to an end. Viruses.

[56] Ayllon J, García-Sastre A, Hale BG (2012). Influenza a viruses and PI3K: are there time, place and manner restrictions?. Virulence.

[57] Elbahesh H, Cline T, Baranovich T, Govorkova EA, Schultz-Cherry S, Russell CJ (2014). Novel roles of focal adhesion kinase in cytoplasmic entry and replication of Influenza A Viruses. J Virol.

[58] Zhang J, Ruan T, Sheng T, Wang J, Sun J, Wang J (2019). Role of c-Jun terminal kinase (JNK) activation in influenza a virus-induced autophagy and replication. Virology.

[59] Kibenge FSB, Kibenge MJT, Kibenge FSB, Godoy MG (2016). Orthomyxoviruses of Fish. Aquaculture Virology.

[60] Ingelfinger D, Arndt-Jovin DJ, Lührmann R, Achsel T (2002). The human LSm1-7 proteins colocalize with the mRNA-degrading enzymes Dcp1/2 and Xrnl in distinct cytoplasmic foci. RNA.

[61] Moon SL, Wilusz J (2013). Cytoplasmic viruses: rage against the (Cellular RNA decay) machine. PLoS Pathog.

[62] Kimura H, Caturegli P, Takahashi M, Suzuki K (2015). New Insights into the function of the Immunoproteasome in Immune and Nonimmune cells. J Immunol Res.

[63] McCarthy MK, Weinberg JB (2015). The immunoproteasome and viral infection: a complex regulator of inflammation. Front Microbiol.

[64] Gervais O, Papadopoulou A, Gratacap R, Hillestad B, Tinch AE, Martin SAM, et al. Transcriptomic response to ISAV infection in the gills, head kidney and spleen of resistant and susceptible Atlantic salmon. bioRxiv. 2022;485193. 10.1101/2022.03.21.485193. 10.1186/s12864-022-09007-4 PMC9703674 36443659

[65] Olsen CM, Markussen T, Thiede B, Rimstad E (2016). Infectious Salmon Anaemia Virus (ISAV) RNA binding protein encoded by segment 8 ORF2 and its Interaction with ISAV and Intracellular Proteins. Viruses.

[66] Rudnicka A, Yamauchi Y (2016). Ubiquitin in influenza virus entry and innate immunity. Viruses.

[67] Gu H, Fada BJ (2020). Specificity in ubiquitination triggered by virus infection. Int J Mol Sci.

[68] Huang X, Wei S, Ni S, Huang Y, Qin Q (2018). Ubiquitin–proteasome system is required for efficient replication of singapore grouper iridovirus. Front Microbiol.

[69] Gack MU, Shin YC, Joo CH, Urano T, Liang C, Sun L (2007). TRIM25 RING-finger E3 ubiquitin ligase is essential for RIG-I-mediated antiviral activity. Nature.

[70] van der Aa LM, Levraud J-P, Yahmi M, Lauret E, Briolat V, Herbomel P (2009). A large new subset of TRIM genes highly diversified by duplication and positive selection in teleost fish. BMC Biol.

[71] Refolo G, Vescovo T, Piacentini M, Fimia GM, Ciccosanti F (2020). Mitochondrial interactome: a Focus on Antiviral Signaling Pathways. Front Cell Dev Biol.

[72] Lei Y, Moore CB, Liesman RM, O’Connor BP, Bergstralh DT, Chen ZJ (2009). MAVS-mediated apoptosis and its inhibition by viral proteins. PLoS ONE.

[73] Tsai C-F, Lin H-Y, Hsu W-L, Tsai C-H (2017). The novel mitochondria localization of influenza a virus NS1 visualized by FlAsH labeling. FEBS Open Bio.

[74] Pila-Castellanos I, Molino D, McKellar J, Lines L, Da Graca J, Tauziet M (2021). Mitochondrial morphodynamics alteration induced by influenza virus infection as a new antiviral strategy. PLoS Pathog.

[75] Svingerud T, Holand JK, Robertsen B (2013). Infectious salmon anemia virus (ISAV) replication is transiently inhibited by Atlantic salmon type I interferon in cell culture. Virus Res.

[76] Jørgensen SM, Hetland DL, Press CM, Grimholt U, Gjøen T (2007). Effect of early infectious salmon anaemia virus (ISAV) infection on expression of MHC pathway genes and type I and II interferon in Atlantic salmon (Salmo salar L.) tissues. Fish Shellfish Immunol.

[77] Liu SY, Aliyari R, Chikere K, Li G, Marsden MD, Smith JK (2013). Interferon-inducible cholesterol-25-hydroxylase broadly inhibits viral entry by production of 25-hydroxycholesterol. Immunity.

[78] Li Y, Anderson DH, Liu Q, Zhou Y (2008). Mechanism of influenza a virus NS1 protein interaction with the p85beta, but not the p85alpha, subunit of phosphatidylinositol 3-kinase (PI3K) and up-regulation of PI3K activity. J Biol Chem.

[79] Momose F, Basler CF, O’Neill RE, Iwamatsu A, Palese P, Nagata K (2001). Cellular splicing factor RAF-2p48/NPI-5/BAT1/UAP56 interacts with the influenza virus nucleoprotein and enhances viral RNA synthesis. J Virol.

[80] Kawaguchi A, Momose F, Nagata K (2011). Replication-coupled and host factor-mediated encapsidation of the influenza virus genome by viral nucleoprotein. J Virol.

[81] Walsh D, Metthews MB, Mohr I (2013). Tinkering with translation: protein synthesis in virus-infected cells. Cold Spring Harb Perspect Biol.

[82] Li S (2019). Regulation of ribosomal proteins on viral infection. Cells.

[83] Dong H-J, Zhang R, Kuang Y, Wang X-J (2021). Selective regulation in ribosome biogenesis and protein production for efficient viral translation. Arch Microbiol.

[84] Tripathi S, Batra J, Cao W, Sharma K, Patel JR, Ranjan P (2013). Influenza a virus nucleoprotein induces apoptosis in human airway epithelial cells: implications of a novel interaction between nucleoprotein and host protein clusterin. Cell Death Dis.

[85] Aviner R, Frydman J (2020). Proteostasis in viral infection: unfolding the Complex Virus-Chaperone interplay. Cold Spring Harb Perspect Biol.

[86] Wen W, Li X, Yin M, Wang H, Qin L, Li H (2021). Selective autophagy receptor SQSTM1/ p62 inhibits Seneca Valley virus replication by targeting viral VP1 and VP3. Autophagy.

[87] Liu S, Mok BW-Y, Deng S, Liu H, Wang P, Song W (2021). Mammalian cells use the autophagy process to restrict avian influenza virus replication. Cell Rep.

[88] Zhang R-h, Zhang H-l, Li P-y, Li C-h, Gao J-p, Li J (2021). Autophagy is involved in the replication of H9N2 influenza virus via the regulation of oxidative stress in alveolar epithelial cells. Virol J.

[89] Wang R, Zhu Y, Zhao J, Ren C, Li P, Chen H (2019). Autophagy promotes replication of Influenza A Virus in Vitro. J Virol.

[90] Kirkin V, McEwan DG, Novak I, Dikic I (2009). A role for ubiquitin in selective autophagy. Mol Cell.

