# Supplementary figures and images for: Understanding host response to infectious salmon anaemia virus in an Atlantic salmon cell line using single-cell RNA sequencing
Source: BMC Genomics. 2023 Mar 29;24:161. doi: 10.1186/s12864-023-09254-z (PMC10061729; doi:10.1186/s12864-023-09254-z)

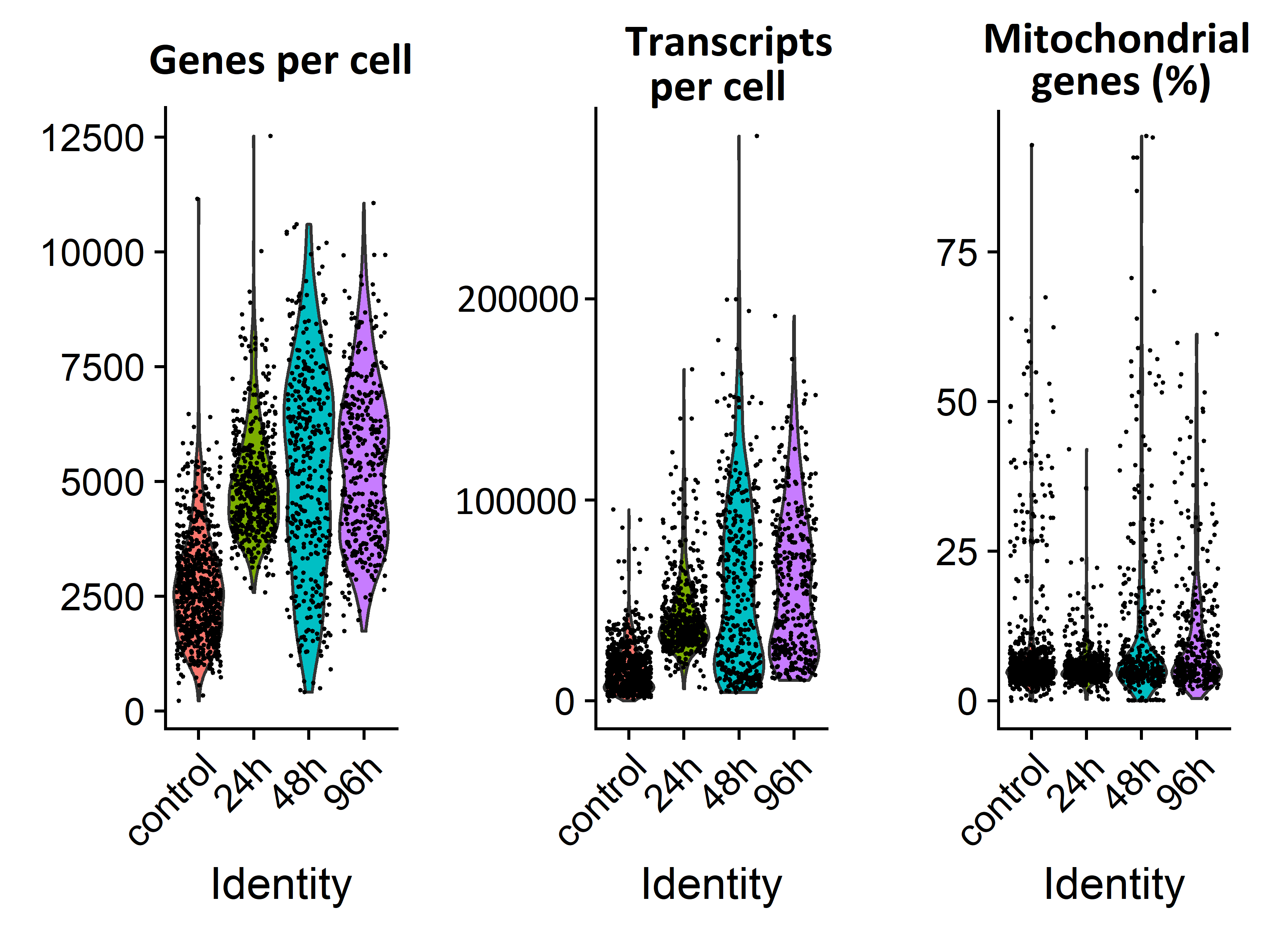

Supplement: Supplementary file 1 — Supplementary Material 1 [file 12864_2023_9254_MOESM1_ESM.png]

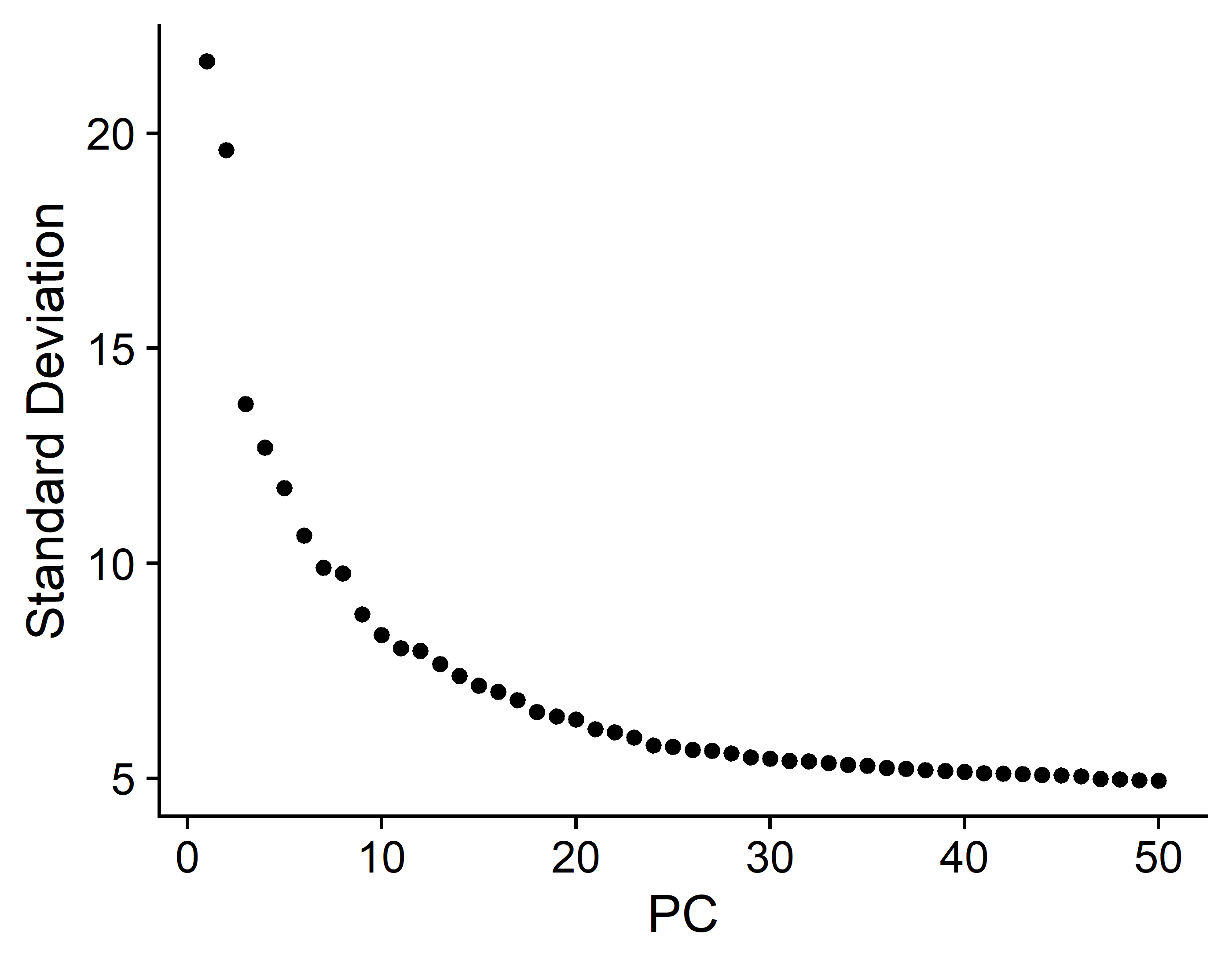

Supplement: Supplementary file 2 — Supplementary Material 2 [file 12864_2023_9254_MOESM2_ESM.png]
