# Supplementary material for: Understanding host response to infectious salmon anaemia virus in an Atlantic salmon cell line using single-cell RNA sequencing
Source: BMC Genomics. 2023 Mar 29;24:161. doi: 10.1186/s12864-023-09254-z (PMC10061729; doi:10.1186/s12864-023-09254-z)
Supplement: Supplementary file 7 — Supplementary Material 7 [file 12864_2023_9254_MOESM7_ESM.docx]

**Supplementary Material**

Supplementary File 1

Excel (.xlsx)

Control vs 24h, 48h and 96h uninfected cells

Differential expression between the cells in the control sample and the uninfected cells of the 24h, 48h and 96h samples.

Supplementary File 2

Excel (.xlsx)

Cluster markers

Genes expressed specifically in each cluster of cells, as defined in Figure 3A

Supplementary File 3

Excel (.xlsx)

KEGG enrichment

KEGG pathways enriched in each cluster of cells, as defined in Figure 3A

Supplementary File 4

Excel (.xlsx)

Correlation host-virus

Correlation between the expression of Atlantic salmon and viral genes in the single-cell RNA-seq dataset

Supplementary Figure 1

Image (.png)

Single-cell RNA-Seq QC

Quality control of the single-cell RNA sequencing data, especially number of genes identified per cell, number of transcripts detected per cell and percentage of mitochondrial genes per cell

Supplementary Figure 2

Image (.png)

Elbow plot

Elbow plot showing the percentage of variation explain by each principal component. The first 30 principal components were used.
